# Data completeness and consistency in individual medical records of institutional births: retrospective crossectional study from Northwest Ethiopia, 2022

**DOI:** 10.1186/s12913-023-10127-0

**Published:** 2023-10-31

**Authors:** Biniam Kefyalew Taye, Lemma Derseh Gezie, Asmamaw Atnafu, Shegaw Anagaw Mengiste, Binyam Tilahun

**Affiliations:** 1 Department of Health Informatics, Institute of Public Health, College of Medicine and Health Sciences, University of Gondar, Gondar, Ethiopia; 2 Ministry of Health, The Federal Democratic Republic of Ethiopia Addis Ababa, Ethiopia; 3 Department of Epidemiology and Biostatistics, Institute of Public Health, College of Medicine and Health Sciences, University of Gondar, Gondar, Ethiopia; 4 Department of Health System and Policy, Institute of Public Health, College of Medicine and Health Sciences, University of Gondar, Gondar, Ethiopia; 5 Management Information Systems, University of South-Eastern Norway, Drammen, Norway

**Keywords:** Completeness, Consistency, Data quality, Institutional birth, Ethiopia, Individual medical records

## Abstract

**Background:**

Ensuring the data quality of Individual Medical Records becomes a crucial strategy in mitigating maternal and newborn morbidity and mortality during and around childbirth. However, previous research in Ethiopia primarily focused on studying data quality of institutional birth at the facility level, overlooking the data quality within Individual Medical Records. This study examined the data completeness and consistency within Individual Medical Records of the institutional birth service and associated factors.

**Methods:**

An institution-based retrospective cross-sectional study was conducted in two districts of Northwest Ethiopia. Data were obtained by reviewing three sets of Individual Medical Records of 651 women: the delivery register, Integrated Individual Folder, and integrated card. The proportions of completeness and consistency were computed. A multilevel binary logistic regression was used to identify factors of completeness and consistency. An odds ratio with a 95% confidence interval was used to assess the level of significance.

**Results:**

Overall, 74.0% of women’s Individual Medical Records demonstrated good data completeness ( > = 70%), 95%CI (70.5, 77.3), while 26% exhibited good consistency, 95%CI (22.9, 29.7). The presence of trained providers in data quality (AOR = 2.9, 95%CI: (1.5, 5.7)) and supportive supervision (AOR = 11.5, 95%CI: (4.8, 27.2)) were found to be associated with completeness. Health facilities’ practice of root cause analysis on data quality gaps (AOR = 8.7, 9%CI: (1.5, 50.9)) was statistically significantly associated with the consistency.

**Conclusions:**

Most medical records were found to have good completeness, but nearly only a quarter of them found to contain consistent data. Completeness and consistency varied on the type of medical record. Health facility’s root cause analysis of data quality gaps, the presence of trained providers in data quality, and supportive supervision from higher officials were identified as factors affecting data quality in institutional birth service. These results emphasize the importance of focused efforts to enhance data completeness and consistency within Individual Medical Records, particularly through consideration of Individual Medical Records in future provider training, supervision, and the implementation of root cause analysis practices.

**Supplementary Information:**

The online version contains supplementary material available at 10.1186/s12913-023-10127-0.

## Background

Poor healthcare quality during and around the time of birth in healthcare facilities becomes a paramount contributor to the persistence of maternal and newborn morbidity and mortality [1]. Quality medical records are emphasized among crucial approaches in improving the quality of care during this critical period [2]. Maintaining quality Individual Medical Records (IMRs) for women is pivotal [3], as these records facilitate early detection and effective management of pregnancy-related complications [4–6]. The World Health Organization (WHO) underscores the importance of quality medical records during and around pregnancy, stating, “Every woman and newborn has a complete, accurate, standardized medical record during labour, childbirth, and the early postnatal period [7].” Quality IMRs also hold a vital role in ensuring the continuity of health services throughout pre-pregnancy to postnatal care [8–12]. They surpass providers’ memory [13], serving as the principal repository of historical health information, thus enhancing patient care quality [14].

In evaluating medical record data quality, two vital dimensions are completeness and consistency [15]. Completeness measures missing data in a dataset [16], while consistency assesses agreement of data across sources like departments, databases, times, or locations [17].

Prior research findings on institutional birth data quality have shown varying levels of completeness and consistency. Studies in India, Ghana, and Ethiopia showed good completeness in institutional birth records, ranging from 75 to 95% [2, 18–20]. Conversely, research in Iraq, Indonesia, and West Africa revealed lower completeness rates, ranging between 22% and 25% [21–23]. Regarding consistency, a study in Rwanda reported a high (98%) consistency rate when comparing institutional birth reports to facility registers [24]. Some studies from Ethiopia demonstrated that institutional birth data consistency ranges from 66 to 97% [25, 26].

In Ethiopia, though prior research has examined the completeness and consistency of institutional birth data, it has predominantly concentrated on evaluating restricted data sources. Completeness was often evaluated in limited medical records like delivery registers [19, 27]. Some studies assessed the completeness of content in reports [28, 29]. Likewise, consistency measurement was frequently involved in comparing aggregate data among the report and recount from the delivery register [25, 26]. Previous studies have not sufficiently addressed the data quality of IMRs in institutional birth. Hence, this study evaluated the IMRs of institutional birth data in two districts in Northwest Ethiopia, aiming to answer the following questions: (1) How is the completeness and consistency of data within IMRs of institutional birth? (2) What factors are associated with the completeness and consistency of data within IMRs of institutional birth?

## Methods

### Study setting

This study was conducted in the “Wegera” and “Tach-armachiho” districts of Central Gondar Zone, Amhara National Regional State, Northwest Ethiopia. The capital of “Wegera” district, “Amba-Giorgis,” is located 232 km from Bahirdar, the capital city of Amhara National Regional State, while the capital of “Tach-armachiho,” “Sanja,” is 298 km from Bahirdar [30]. In 2021, the total population in the two districts was 398,350 [31]. The population of women in reproductive age was estimated at 93,214 (23.4%) [32]. Two primary hospitals, 13 health centers, and 72 health posts provide comprehensive health services for the two districts’ population.

The study sites were chosen through a joint decision involving the Central Gondar Zone, Amhara Region Health Bureau, and the University of Gondar. Both districts are currently participating in the Capacity Building and Mentoring Partnership (CBMP) program, in collaboration with the Ethiopian Ministry of Health(FMOH) and the University of Gondar, aimed at enhancing health data quality and utilization [33]. The University of Gondar offers technical support, including training, supervision, and mentorship, to local health facilities as part of CBMP intervention [34].

#### Existing institutional birth data management

Ethiopia is implementing the routine Health Management Information System (HMIS), which has been in place since 2008 [35]. The HMIS was revised in 2017 to enhance the monitoring of priority health outcomes [36]. Institutional birth is a core indicator of the revised HMIS and Ethiopia’s Health Sector Transformation Plan (HSTP-II), guiding decision-making across all healthcare system levels to mitigate the country’s persistent maternal and newborn mortality challenges [37]. In Ethiopian HMIS, the data source for institutional birth coverage is mainly from governmental institution-based medical records [38]. According to the Ethiopian data recording and reporting procedures guideline [39], the central medical records designed for the documentation of institutional birth service are three: (1) The delivery register, (2) The women’s Integrated Individual Folder (IIF), and (3) the ‘Integrated Antenatal, Delivery, Newborn, and Postnatal Care Card’ – the integrated card hereafter.

The *delivery register* captures data for each woman giving birth, including identification, services received, and health outcomes for both mother and newborn. Each row in the delivery register corresponds to the data of a particular woman who has given birth at healthcare facilities. It is typically stored in the Maternal and Child Health Department (MCH), where institutional birth occurs. The *Integrated Individual Folder* serves to record mainly the women’s identity details and other socio-demographic variables of women. It also functions as a pouch to contain all other IMRs of women, including the integrated card. The *integrated card* gathers data for women throughout Ante Natal Care (ANC), intrapartum, labor, delivery, and Post Natal Care (PNC).

Both the IIF and integrated card are archived in the Medical Record Unit(MRU). After data capture by providers at the MCH, the integrated card must be promptly filed within the women’s IIF at the MRU [39].

### Study design and period

An institution-based retrospective cross-sectional survey was done to examine the completeness and consistency of the IMRs pertaining to institutional birth. The data collection for this study was done in late December 2021 for 12 days (17–28/December 2021).

### Study subjects and sample size

The study subjects of this study consisted of the three pairs of IMRs(the delivery register, IIF, and the integrated card) of women who gave birth within the six months preceding the survey. These three sets of IMRs were paired together using the mother’s Medical Record Number (MRN) to form a single study subject.

The required sample size was determined using a single population proportion formula:

$$n=\frac{\left(Z{\displaystyle_{a/2}}\right)^2.pq}{d^2}$$

Where *n* = the initial sample size.

z_α/2_= reliability coefficient at 95% confidence level = 1.96, (z_α/2_)^2^ = 3.8416.

*p* = proportion of outcomes (data completeness and consistency).

q = 1-p.

d = margin of error = 0.05, and thus d^2^ = 0.0025.

Considering completeness and consistency as this study’s outcomes, we have 80.3% completeness and 29.5% consistency, obtained from a pilot study conducted prior to this main study in the “Gondar zuria” district, Northwest Ethiopia. These proportions were assumed to compute the required sample, with additional assumptions of a design effect of 2 and a 10% non-response rate. The computed sample sizes were: ≈535 based on the completeness proportion and ≈ 703 based on the consistency proportion. Consequently, the largest sample size of 703, which was computed using the proportion of consistency, was used for this study.

### Sampling procedure

**Fig. 1:**
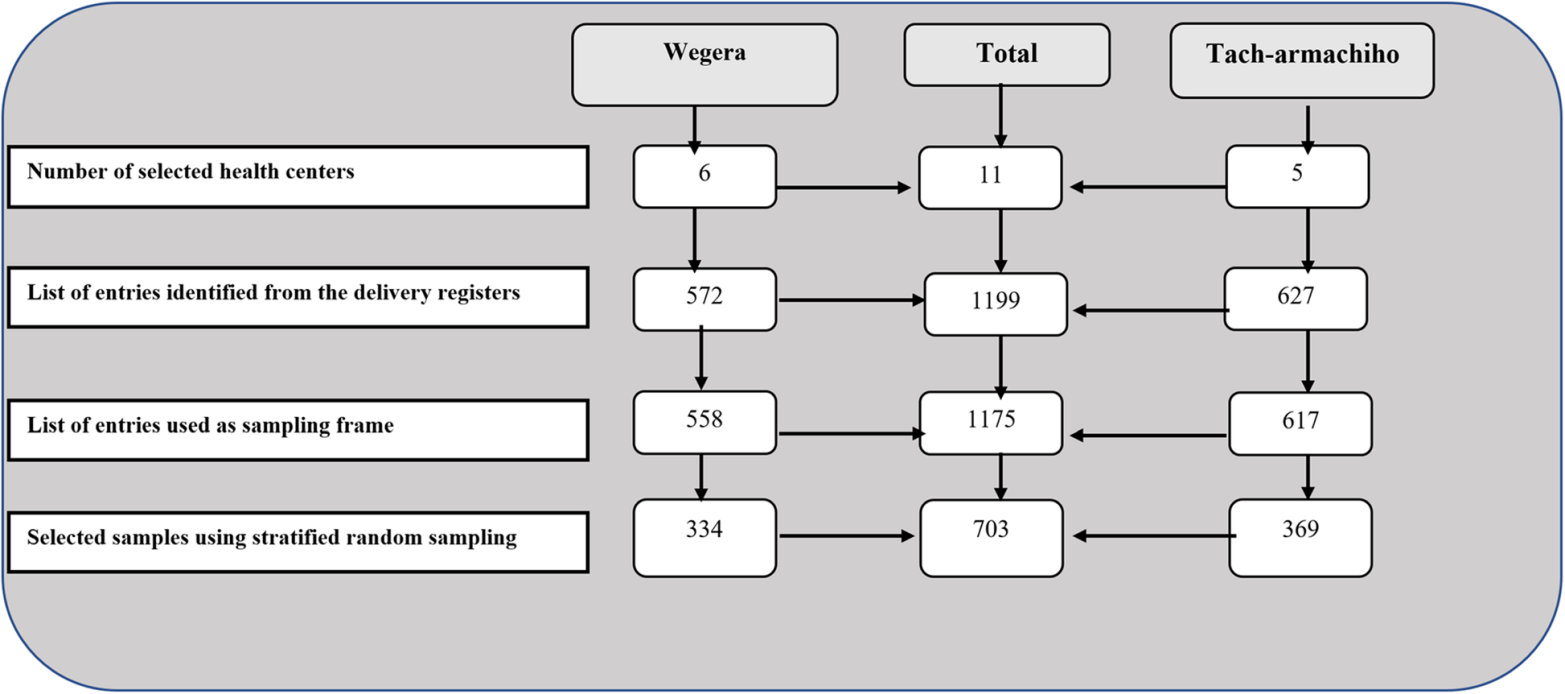
Diagram showing selection procedure of study samples

The “Wegera” and “Tach-armacheho”districts had eight and five health centers, respectively. We selected six health centers in “Wegera” and five(all) in “Tach-armacheho” districts, giving a total of eleven health centers. The samples in this study were selected from a list of entries(rows) in the delivery register. Six months before the survey date, we identified 1199 entries of women who had delivered at the selected health centers in the two districts. The inclusion criteria for selecting the study subjects was the readability of women’s MRN in the delivery register. According to the guideline [39], each woman would have a unique MRN to facilitate the identification and matching of IMRs. Therefore, women’s entries with readable MRNs were necessary to match the three sets of IMRs targeted by this study. Accordingly, of the 1199 women’s entries on the delivery register,1175 had readable MRNs. These 1175 entries of women were used as a sampling frame, and samples were selected using Stratified Random Sampling (Fig. 1).

### Variables and measurement

#### Outcome variables

##### Completeness

This study adopted a completeness definition proposed in previous study [17], where completeness refers to the presence of all required data elements in a target dataset. Thus, we assessed completeness by reviewing the presence of data elements that are expected to be contained in the delivery register, IIF, and integrated card. Of the data elements on these IMRs, completeness was determined for data elements mandatory for recording. The IMRs considered in this study did not require obligatory recording for some data elements. For instance, we excluded the mother’s HIV test from the completeness assessment since incomplete HIV data could be due to the absence of HIV services, rather than missing information. Incomplete data may not imply missing; that missing could simply result from an event that did not occur, as indicated by some previous studies [40]. Such data elements were not included to measure completeness in this study. Accordingly, completeness was analyzed for 33 variables in total. Of the 33 variables, 10 were from the delivery register, 7 were from the IIF, and 16 were from the integrated card.

##### Consistency

We evaluated consistency by cross-checking the data element’s agreement across the IMRs [41]. The data elements documented in the delivery register were verified for agreement with both the IIF and the integrated card. The delivery register was considered the gold standard due to its inclusion of a comprehensive set of shared data elements, allowing for comparison with the IIF and integrated card. Accordingly, two data elements, a “serial number” and “date of delivery,“ were extracted from the delivery register and compared to the data fields in the woman’s IIF. To examine the consistency between the delivery register and the integrated card, we compared the IMRs of women on 11 data elements (“MRN”, “name of mother”, “date of delivery”, “time of delivery ”, “mode of delivery”, “sex of newborn”, “name and signature of provider”, “Apgar score”, “newborn weight”, “women’s HIV test accepted”, and “women’s HIV test result”).

#### Independent variables

We measured and incorporated the following independent variables in our analysis to identify factors associated with the completeness and consistency of data within IMRs: 

##### The presence of health information technology (HIT)

This variable determined the existence of a designated person responsible for institutional birth data entry and report compilation, coded as ‘1’ (‘Yes’) for presence and ‘0’ (‘No’) for absence.

##### Availability of data recording tools

Assessed the availability of HMIS data recording tools over six months, coded ‘1’ (‘Yes’) for uniform availability throughout the six months and ‘0’ (‘Partially available’) otherwise.

##### Availability of trained providers

Measured staff training on institutional birth data review and quality, coded ‘1’ (‘Mostly trained’) if all staff has received training in the past six months and ‘0’ (‘Partially trained’) if some staff have received training in the past six months.

##### Supportive supervisions from higher officials

Measured the extent of supportive supervision from higher-level officials over the past six months before the survey, coded ‘1’ (‘At least three times’) for at least three visits and ‘0’ (‘Less than three times’) for fewer.

##### Existence of Performance Monitoring Team(PMT)

Determined PMT establishment, coded ‘1’ (‘Yes’) for presence and ‘0’ (‘No’) for absence.

##### The PMT per membership standard

Assessed PMT members composition, coded ‘1’ (‘Yes’) for standard membership composition (the team comprised of the Head of the institution, HMIS in charge, and all representatives of the case teams) and ‘0’ (‘No’) otherwise.

##### Monthly PMT meeting in the last 6 months

Measured monthly PMT meetings supported with minutes, coded ‘1’ (‘Yes’) for regularity (conducted each month) and ‘0’ (‘No’) otherwise.

##### Monthly conducted lot quality assurance sampling (LQAS) in the last 6 months

Evaluated monthly LQAS reviews supported with minutes, coded ‘1’ (‘Yes’) for regularity (conducted each month) and ‘0’ (‘No’) otherwise.

##### Conducted Root Cause Analysis (RCA) on identified gap

Determined RCA performance on the identified data quality gaps, coded ‘1’ (‘Yes’) for conducted RCA and ‘0’ (‘No’) for absence.

##### Internal supervisions

Measured internal supervisory visits by the management of the health facilities, coded ‘1’ (‘At least two times’) for at least two visits and ‘0’ (‘Less than two times’) for fewer.

##### Availability of HMIS guidelines

Assessed guideline presence, coded ‘1’ (‘Yes’) for all guidelines available (Health Data quality, HMIS Procedure manual, Indicator reference manual, and Information Use manual) and ‘0’ (‘Partially available’) for any missing guideline.

### Data collection

The IMRs were reviewed using a checklist developed by researchers in this study (Supplementary 1). A checklist was prepared following pertinent Routine Health Information System(RHIS) literature, including the Performance of Routine Information System Management (PRISM) framework [42, 43] and Ethiopian HMIS-related guidelines [36, 39, 44]. A checklist was piloted in the “Gondar Zuria” district on 78 study participants (11% of the study sample). Cronbach's alpha was computed to evaluate the internal consistency reliability of the items of the outcome variables, revealing a better reliability of 0.86. Eleven data collectors, including HIT personnel and relevant health sciences graduates, were recruited to collect the data. Data collectors were recruited from district health offices and local health facilities within the study sites. The data collectors received three days of training regarding the study's objectives, methodologies, and ethical considerations.   The principal investigator and the other two deployed supervisors closely supervised the entire data collection.

### Data analysis

Data were entered into Epi-data v-3.1 and analyzed using the statistical software Stata v-17.0. We calculated the frequency and percentage of completeness and consistency for data elements and IMR type.

Concerning completeness, each data element was coded as 1"Yes" if recorded and 0"No" if unrecorded for each study subject. The completeness proportion was determined for data elements by dividing the total number of study subjects with recorded data elements by the total sample size. The mean completeness rates were separately computed for the three IMRs by adding the count of recorded data elements for each study subject and then dividing it by the respective total expected number of data elements. The overall average completeness proportion was determined by summing the recorded data elements per study subject and dividing this sum by 33(the total expected number of data elements across the three IMRs).

Similarly, for consistency, data elements were coded as 1"Yes" if consistently recorded and 0"No" if not consistently recorded. The consistency proportion per data element was calculated by dividing the sum of subjects with consistent data elements by the total sample. The average consistency rate was calculated by adding up the number of consistently recorded data elements per woman's IMRs and dividing it by the total number of expected data elements.

We applied the WHO's minimum acceptable data quality threshold of 70% [45] to categorize completeness and consistency as "Good" or "Poor." As a result, we categorized completeness as "Good" if the set of IMRs had 70% or more complete data and as "Poor" otherwise. Similarly, we categorized consistency as "Good" if the set of IMRs contained 70% or more consistent data and as "Poor" otherwise.

#### Multilevel logistic regression analysis

Our outcome variables, completeness and consistency, were defined at level one, at the level of the study subjects (pairs of women's IMR). Given that study subjects are nested within the health centers they belong to, we assume more similarities among subjects within the same health center regarding this study's outcome variables. The intra-class correlation coefficients (ICC) were calculated to determine the degree of similarity of study subjects within health centers (clusters) [46, 47]. Unlike outcome variables, all the independent variables in our study were measured at the health center's level (level two). However, owing to the flexibility inherent in multilevel modeling [47], we employed a disaggregation approach to assign values of independent variables to each study unit for the multilevel regression analysis.

Accordingly, to examine the association between the independent variables and outcome variables, a multilevel (two level) binary logistic regression model was employed. We computed crude and adjusted Odds Ratios using two-level binary logistic regression and considered a corresponding 95% CI to determine the statistical significance and precision of the point estimate. Hosmer-Lemeshow goodness fit statistic was used for the model fitness [48]. Variance Inflation Factor (VIF) was used to assess multicollinearity, and VIF > 5 was considered to declare the multicollinearity of the independent variables [49]. To address multicollinearity, some independent variables were excluded from the final model. Receiver-operating characteristics (ROC) were calculated to measure the discriminative power of the models [50].

## Results

### Description of women’s IMRs by health facilities characteristics

Data analysis was performed on 651 (92.6%) study subjects with three IMR types: delivery register, integrated card, and IIF. The distribution of study subjects was similar across most of the health facilities’ characteristics. Frequency variations were observed with the following variables: the presence of trained providers in data quality, availability of the data recording tools, number of supportive supervisions from the higher officials, availability of HMIS guidelines, performance of internal supervision, and health facilities’ RCA on data quality gaps.

The majority,598(91.9%) of the IMRs were from health centers with HMIS guidelines. More than half, 452(69.4%) of the IMRs were from the health centers where most providers received training on data quality. Less than half, 293(45.0%) of IMRs were from the health centers where the data recording tools partially existed. Nearly half, 330(50.7%) of the IMRs were from health centers with at least three supportive supervision visits from higher officials six months before the study period (Table 1).

**Table 1:** Women’s IMR distribution by health center characteristics in two districts of Northwest Ethiopia, 2022

Variables	Number of medical records (*n*=651)	Percentage
Presence of HIT	651	100.0
Availability of data recording tools		
Yes available	358	54.9
Partially available	293	45.0
Availability of trained providers		
Partially	199	30.6
Mostly	452	69.4
Supportive supervisions from higher officials		
Less than 3-times	321	49.3
At least 3-times	330	50.7
Existence of PMT	651	100
The PMT per membership standard	651	100
Monthly PMT meeting in the last 6 months	651	100
Monthly conducted LQAS in the last 6 months	651	100
Conducted RCA on identified gap		
No	364	55.9
Yes	287	44.1
Internal supervision		
Less than 2-times	549	84.3
At least 2-times	102	15.7
Availability of HMIS guidelines		
Partially available	53	8.10
Yes available	598	91.9

### Completeness of institutional birth data

#### Average completeness rate by type of IMRs

**Fig. 2:**
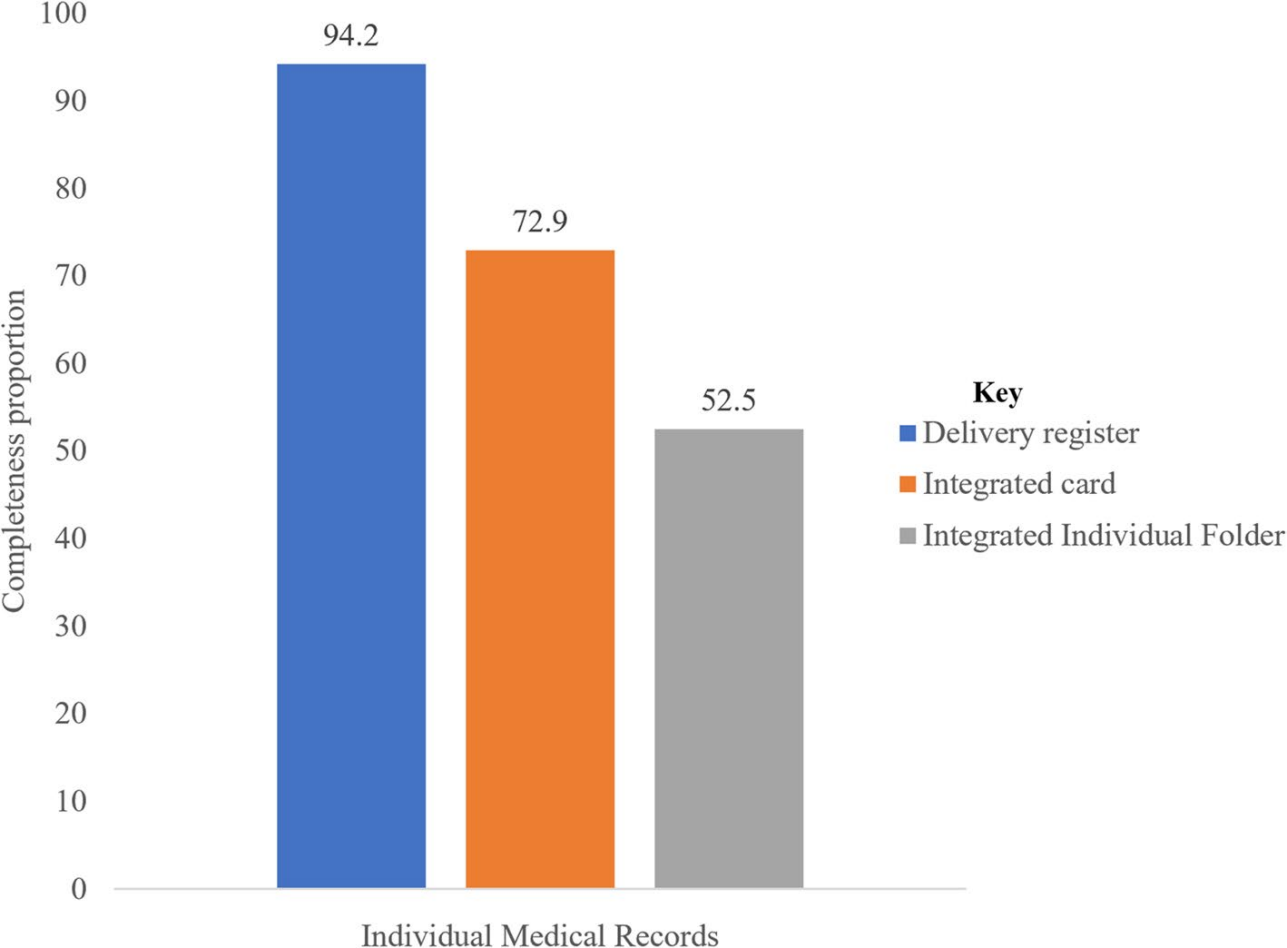
Completeness of institutional birth data, by IMRs type in two districts of Northwest Ethiopia, 2022

On average, the delivery register contained 94.2% complete data, 95%CI (93.0, 94.1). In comparison, integrated cards contained 72.9% complete data (95% CI: 70.1, 75.9), and the IIF showed the lowest completeness score at 52.5% (95% CI: 50.5, 54.1). The mean completeness rate across the three IMRs was 78.1%, 95%CI (76.5, 79.8). The data completeness for 74.0% of IMRs was good ( > = 70%), 95%CI (70.5, 77.3) (Fig. 2).

#### Completeness of the specific institutional birth data elements

Regarding the data elements in the delivery register, the “Serial Number” was completely recorded for all study subjects. Except for the “name and signature of providers,” showed 78.9% completeness (95% CI: 75.8, 82.1), and the “time of delivery” showed 64.1% completeness (95% CI: 60.4, 67.8), all other variables in the delivery register showed completeness rates over 90%.

The “Serial Number” in the IIF showed a completeness rate of 9.5% (95% CI: 7.30, 11.8). In the IIF, the lowest completeness rates were observed for two other variables: the “name of the department” with 9.8%(95% CI: 7.50, 12.1) and the “date of delivery” with 9.7% (95% CI: 7.40, 11.9). However, it was found that the completeness rates for the remaining data elements in the IIF exceeded 76%.

Concerning the data elements from the Integrated Card, this study revealed completeness rates ranged from 57.1 to 78.9%. While “Name of Mother” and “Gravida” showed completeness rates of 78.9%(95% CI: 75.8, 82.1) and 72.6%(95% CI: 69.2, 76.1) respectively, “Ruptured Membranes” was recorded at a lower rate of 57.1%(95% CI: 53.3, 60.9) (Table 2).

**Table 2:** Completeness of the specific institutional birth data elements in two districts of Northwest Ethiopia, 2022

Variables	Recorded (n)	Percentage (95%CI)
**Delivery register**		
Serial number	651	100
Age	644	98.9 (98.1, 99.7)
Address	645	99.1 (98.3, 99.8)
Date of delivery	643	98.8 (97.9, 99.6)
Time of delivery	417	64.1 (60.4, 67.8)
Newborn outcome	647	99.4 (98.8, 99.9)
Mode of delivery	643	98.8 (97.9, 99.6)
Maternal status	647	99.4 (98.8, 99.9)
Sex of newborn	641	98.5 (97.5, 99.4)
Name and signature of provider	514	78.9 (75.8, 82.1)
**IIF**		
Name of facility	497	76.3 (73.1, 79.6)
Date of registration	569	87.4 (84.8, 89.9)
Sex of client	587	90.2 (87.8, 92.5)
Age of mother	542	83.2 (80.3, 86.1)
Date of delivery	63	9.70 (7.40, 11.9)
Name of the department	64	9.80 (7.50, 12.1)
Serial number	62	9.50 (7.30, 11.8)
**Integrated card**		
Name of mother	514	78.9(75.8, 82.1)
Gravida	473	72.6(69.2, 76.1)
Para	502	77.1(73.9, 80.3)
MRN	462	70.9(67.5, 74.5)
Date of Admission	502	77.1(73.9, 80.3)
Time of Admission	478	73.4(70.0, 76.8)
Ruptured Membranes	372	57.1(53.3, 60.9)
Date of delivery	506	77.7(74.5, 80.9)
Time of delivery	485	74.5(71.1, 77.9)
Mode of delivery	463	71.1(67.6, 74.6)
Placenta	477	73.3(69.9, 76.7)
Sex of newborn	488	74.9(71.6, 78.3)
Newborn outcome	496	76.2(72.9, 79.5)
Single-multiple	471	72.4(68.9, 75.8)
Term-preterm	462	70.9(67.5,74.5)
Name and signature of providers	448	68.8(65.2,72.4)

### Consistency of institutional birth data across IMRs

The average consistency among IMRs was estimated at 55.2%, 95%CI (53.2, 57.2). Twenty- six percent of IMRs exhibited good consistency ( > = 70%), 95%CI (22.9, 29.7).

Among the delivery register and IIF, the “Serial number” demonstrated a consistency rate of 4.8% (95% CI: 3.10, 6.40), while the “Date of delivery” exhibited a consistency rate of 6.7% (95% CI: 4.70, 8.60).

Across the comparison between the delivery register and integrated card, the consistency rates varied for different variables: “Newborn weight” showed a high consistency rate of 80.7% (95% CI: 77.6, 83.7), the “name and signature of provider” had a consistency rate of 56.1% (95% CI: 52.2, 59.9), “Time of delivery” was consistently recorded in 44.1% of records (95% CI: 40.3, 47.9), while just 23.5% of records displayed consistent HIV testing acceptance of women (95% CI: 20.3, 26.8) (Table 3).

**Table 3:** Consistency of institutional birth data across IMRs in two districts of Northwest Ethiopia, 2022

Variable	Consistently recorded (n)	Percentage (95%CI)
**Consistency among delivery register and IIF**		
Serial number	31	4.8(3.10, 6.40)
Date of delivery	43	6.7(4.70, 8.60)
**Consistency among delivery register and integrated card**		
MRN	462	70.9(67.5, 74.5)
Name of mother	514	78.9(75.8, 82.1)
Date of delivery	502	77.1(73.9, 80.3)
Time of delivery	287	44.1(40.3, 47.9)
Mode of delivery	456	70.1(66.5, 73.6)
Sex of newborn	481	73.9(70.5, 77.3)
Name and signature of provider	365	56.1(52.2, 59.9)
Apgar score	515	79.6(76.5, 82.7)
Newborn weight	522	80.7(77.6, 83.7)
Women’s HIV test accepted	153	23.5(20.3, 26.8)
Women’s HIV test result	48.5(44.6, 52.3)	314

### Factors associated with completeness and consistency of institutional birth data

In multilevel binary logistic regression, after adjusting for other factors, health facilities with mostly trained providers showed a 2.9-fold increase (AOR = 2.9, 95% CI: 1.5, 5.7) in the likelihood of containing good complete data recording compared to those with partially trained providers. Similarly, health facilities with at least three times SS were associated with a 11.5-fold increase (AOR = 11.5, 95% CI: 4.8, 27.2) in chance of IMRs containing good data completeness compared to those with less than three times SS. (Table 4).

**Table 4:** Factors associated with completeness of institutional birth data in two districts of Northwest Ethiopia, 2022

Variables	Completeness		Odds ratio with 95% CI	
	Good n (%)	Poor n (%)	Crude odds ratio	Adjusted odds ratio
**Existence of trained providers**				
Partly trained	100(50.3)	99(49.8)	1	1
Mostly Trained	382(84.5)	70(15.5)	8.2 (1.9, 35.7)	2.9 (1.5, 5.7)
**Supportive supervisions from higher officials**				
Less than 3 -times	170(52.9)	151 (47.0)	1	1
At least 3-times	312(94.6)	18 (5.5)	16.8 (7.4, 38.2)	11.5(4.8, 27.2)
**Root cause analysis**				
No	254(69.8)	110 (30.2)	1	1
Yes	228(79.4)	59 (20.6)	2.3(0.25, 205)	1.8(0.9, 3.9)
**Internal supervision**				
Less than 2 times	403(73.4)	146(26.6)	1	1
At least 2 times	79(77.5)	23(22.6)	1.4(0.22, 82.1)	1.9(0.9, 4.3)

*Goodness of fit indices: Prob > chi2 = 0.46; Mean VIF = 3.73; Area under ROC curve = 0.83*

The IMRs from health facilities that conducted the RCA on data quality gaps demonstrated about ninefold higher chance (AOR = 8.7, (95% CI: 1.5, 50.9)) of good consistency compared to IMRs from facilities that did not engage in the RCA practice (Table 5).

**Table 5:** Factors associated with consistency of institutional birth data in two districts of Northwest Ethiopia, 2022

Variables	Consistency		Odds ratio with 95% CI	
	Good n (%)	Poor n (%)	Crude odds ratio	Adjusted odds ratio
**Existence of trained providers**				
Partly trained	56(28.1)	143(71.9)	1	1
Mostly Trained	114(25.2)	338(74.8)	2.3 (0.3,17.3)	3.3(0.6, 17.2)
**Supportive supervisions from higher officials**				
Less than 3 -times	68(21.2)	253(78.8)	1	1
At least 3-times	102 (30.9)	228(69.1)	2.9(0.4, 22.3)	1.5(0.3, 7.5)
**Root cause analysis**				
No	36(9.9)	328(90.1)	1	1
Yes	134(43.7)	153(53.3)	7.8 (1.4,43.6)	8.7(1.5, 50.9)
**Internal supervision**				
Less than 2 times	120(21.9)	429(78.1)	1	1
At least 2 times	50 (49.0)	52(50.9)	2.6(0.3, 18.7)	1.5(0.3, 8.6)

*Goodness of fit indices: Prob > chi2 = 0.2; Mean VIF = 2.3; Area under ROC curve = 0.76*

In the null model (model without independent variables), the variances for completeness and consistency across health centers were estimated at 2.37 and 2.69, respectively. These variance estimates produced an ICC of 0.42 for completeness and 0.45 for consistency. The ICC results showed that variations across health centers explained 42% of the variation in odds of completeness and 45% of the variation in consistency. However, a reduction in ICC was observed when comparing the final models to the null models, with the ICC for completeness decreasing from 42% to nearly 0% and the ICC for consistency decreasing from 45–25% (Table 6).

**Table 6:** ICC estimates of institutional birth data completeness and consistency in two districts of Northwest Ethiopia, 2022

Estimates	Completeness		Consistency	
	Null Model	Final Model	Null Model	Final Model
Between cluster variance	2.37	1.91e-34	2.69	1.13
ICC	0.42	5.80e-35	0.45	0.25

## Discussion

This research examined the completeness and consistency of data within IMRs of institutional birth and associated factors. On average, over three-fourths of the pairs of IMRs were complete, and over half were consistent. The presence of trained providers in data quality and support and supervision from higher officials were the identified statistically significant factors of good data completeness. Health facilities’ RCA on data quality gaps was identified as a predictor of good consistency.

Our study found a mean completeness rate of 78% across three IMRs. This result aligns with previous research in Ethiopia [27], which reported a 78% completeness rate for institutional birth records. Our study’s findings exceed those from a study in Ghana [20] and Tanzania [51], which reported 75% and 68% completeness rates, respectively. Moreover, our result is far higher than that of a study conducted in Southern Ethiopia [52], which reported a completeness rate of only 18.4%. This difference indicates the variability in the completeness of IMR data across contexts and types of health institutions. Our study, specifically focused on data completeness within the context of health centers, may reflect a distinct result compared to the Tanzanian and Southern Ethiopian studies that assessed IMRs within hospital settings. The varying completeness levels observed across different healthcare settings underscore the need to tailor the data completeness enhancement strategies to each healthcare context.

Nonetheless, our study’s finding on completeness is lower than that in Northern Ethiopia, which showed 95% completeness [19]. The discrepancy in findings may be attributed to differences in sample sizes and the range of variables examined among these studies. Notably, our study involved a remarkably larger sample of IMRs compared to the Northern Ethiopian study, which reviewed just 50 records. Besides, our research comprehensively assessed completeness across various data elements within different IMRs. The lower completeness rate identified in our study compared to the previous study implies the need for further efforts to enhance data completeness in institutional birth. Our study suggests the inclusion of thorough IMR assessments in future policies and practices for the betterment of institutional birth and the broader scope of RHIS data quality [53–55].

In this study, the data consistency among IMRs averaged 56%, and only 26% demonstrated good consistency ( > = 70%). These findings deviate from previous studies on the consistency of institutional birth data, as a report from Rwanda [24] indicated a consistency rate of 98%, whereas a study in Southern Ethiopia [25] reported a consistency rate of 96.9%. The disparity in results could result from differences in the approaches used to measure consistency. Previous studies employed the Verification Factor (VF), which involved comparing institutional birth reports with data recounted from the source documents [45]. In contrast, our study compared specific data elements across women’s IMRs. While the conventional VF approach provides valuable insights into data quality at aggregated levels (at the health facility and above levels) [45], understanding data quality within women’s IMRs is vital for delivering high-quality care to individual client’s level [56]. Hence, our study calls for further enhancements of institutional birth data quality and emphasizes the need for a detailed consistency assessment across women’s IMRs [41, 57–59]. Improving data quality in women’s IMRs can greatly assist healthcare providers in identifying and addressing pregnancy-related causes of death, which is vital in reducing maternal and neonatal mortality through effective data utilization [7].

This study revealed that types of IMRs and data elements varied in completeness and consistency. Notably, most women’s IIF lacked quality recording of critical variables, including the “serial number,“ “name of the department,“ and “date of delivery.“ Since these variables were initially intended to guarantee continuity of care throughout pregnancy and childbirth [39], their incomplete and inconsistent recording raises concerns about the quality of maternity care at healthcare facilities. For policymakers and practitioners, our findings emphasize the need to implement strategies that enhance the accurate recording of these essential variables to improve the continuity and quality of pregnancy-related care. Besides, despite some prior research indicating variations in the quality across variables within medical records [60], the reasons behind the lack of quality recording of such essential variables still need to be clarified. Hence, future research needs to prioritize conducting thorough investigations to gain insights into the factors behind variations in data quality across different IMRs and data elements.

This study’s findings revealed a higher likelihood of good completeness in IMRs when facilities are mostly staffed by trained providers in data quality. This finding aligns with a previous study conducted in Ethiopia, which identified a significant association between training and data quality [61, 62]. Regular training is widely acknowledged as a key factor in enhancing providers’ motivation and competence for better performance in data quality and the range of maternity services quality at health facilities [63, 64].

Our study revealed that supportive supervision by higher-level officials is another significant factor linked to the good completeness of data in institutional birth. This finding is consistent with prior studies conducted in Ethiopia and Indonesia [65, 66], which highlighted the effectiveness of supportive supervision in improving health care data quality. Therefore, Policymakers, program managers, and officials at the district and higher administrative levels need to prioritize strengthening regular supervision and directives for healthcare facilities, enabling healthcare providers to promptly identify and correct data quality gaps in the services they deliver [67].

In contrast to data completeness, this study revealed that training and supportive supervision were not significantly associated with data consistency within IMRs of institutional birth. This study’s contrast with previous findings [62, 63, 68] underscores the importance of tailoring data quality improvement efforts in RHIS to address the specific challenges within IMRs. Policymakers and program managers need to consider incorporating IMR-specific training and support into future initiatives to enhance the data quality of institutional birth.

In this study, conducting RCA to address identified data quality gaps was found to have a positive association with the good consistency of institutional birth data.Prior studies have reported the effectiveness of routine data quality evaluation methods like LQAS in ensuring the quality of RHIS data [62, 65]. In our study, although LQAS performance was uniform across health facilities, differences in RCA practice demonstrated its importance in improving the data quality across IMRs in institutional birth. The RCA is effective because it allows healthcare organizations to engage in in-depth discussions regarding the barriers to data quality [69]. Hence, the result of our study emphasizes the importance of establishing clear RCA implementation guidelines and ongoing stakeholder follow-up for maintaining its implementation further to enhance the institutional birth and overall RHIS data quality. In practice, healthcare facilities and providers need to recognize RCA’s value in improving data quality. Emphasizing RCA sessions in the existing HMIS training programs could help healthcare providers value the benefits of RCA in enhancing data quality.

To conclude, unlike prior research, which focused on evaluating data quality at the institutional level, our study assessed data quality within IMRs of institutional births and the associated factors. However, it is essential to note a potential limitation: the inherent subjectivity and potential for interviewer bias when data collectors judge completeness and consistency. To minimize interviewer bias, the purpose of the study was emphasized during the data collection training.

## Conclusions

While consistency fell far short of the standard, most of IMRs exhibited good completeness. The proportions of completeness and consistency varied across the three types of IMRs examined in this study. Factors identified to affect the completeness and consistency of data within IMRs of institutional birth include the presence of trained personnel responsible for data quality, the extent of supportive supervision from higher-level officials, and the practice of RCA within health facilities to address data quality gaps. In light of these findings, it is essential to integrate IMRs into existing RHIS data quality assessment initiatives, strengthen the implementation of RCA practices at the health facility level, and continue offering training and support to healthcare facilities. These essential measures for enhancing the quality of institutional birth data and overall data within the RHIS are particularly crucial in resource-limited settings like Ethiopia.

### Supplementary Information

**Additional file 1: Supplementary 1.** A Checklist for reviewing Individual medical records

## Data Availability

The corresponding author can provide data upon reasonable request.

## Abbreviations

ANC: Antenatal care
COR: Crude odds ratio
AOR: Adjusted odds ratio
CBMP: Capacity Building and Mentorship Program
CI: Confidence interval
FMOH: Ethiopian Federal Ministry of Health
HIT: Health Information Technology
HMIS: Health Management Information System
IIF: Integrated Individual Folder
IMR: Individual Medical Record
LQAS: Lot Quality Assurance Sampling
MCH: Maternal and Child Health Department
MRN: Medical Record Number
MRU: Medical Record Unit
PMT: Performance Monitoring Team
PNC: Post Natal Care
RCA: Root Cause Analysis
RHIS: Routine Health Information System
ROC: Receiver-operating characteristics
SS: Supportive supervision
VIF: Variance Inflation Factor
WHO: World Health Organization
