# Supplementary material for: Data completeness and consistency in individual medical records of institutional births: retrospective crossectional study from Northwest Ethiopia, 2022
Source: BMC Health Serv Res. 2023 Oct 31;23:1189. doi: 10.1186/s12913-023-10127-0 (PMC10619314; doi:10.1186/s12913-023-10127-0)
Supplement: Supplementary file 1 — Additional file 1: Supplementary 1. A Checklist for reviewing Individual medical records [file 12913_2023_10127_MOESM1_ESM.doc]

**Supplementary 1. A Checklist for reviewing Individual medical records**

To obtain the IIF and integrated cards, reach out to the MRU focal person, and for the delivery register, contact the MCH focal person. Otherwise, kindly consult the head of the health centers to designate a suitable person to assist you in locating and reviewing the IMRS.

**Introduction:**

Greetings, and I hope you're having a pleasant morning or afternoon. My name is_________, and I sincerely appreciate your willingness to participate in this discussion today. I am here to gather data as part of a research project by the University of Gondar, specifically focusing on data quality within institutional birth. Your collaboration is vital to the success of this study.

I would appreciate your assistance in providing me with medical records related to institutional birth data. I aim to review each medical record's data elements for quality recordings. If, at any point, you find yourself unable to continue with this process, please do not hesitate to inform us. Can I start an interview?

The participant declined participation;

**___________________ // ___________________ // ___________________**

Name of Participant Date Signature

**___________________ // ___________________ // ___________________**

Name of data collector Date Signature

The participant agreed to participate in the document review

**___________________ // ___________________ // ___________________**

Name of Participant Date Signature

**___________________ // ___________________ // ___________________**

Name of data collector Date Signature

|  | | | | | **Section 1: Completeness of data elements on delivery register** | | | | | | | | | | | | | | | | |  | | | |  | | | | |
| --- | --- | --- | --- | --- | --- | --- | --- | --- | --- | --- | --- | --- | --- | --- | --- | --- | --- | --- | --- | --- | --- | --- | --- | --- | --- | --- | --- | --- | --- | --- |
| 101 | | | | | **Identification /Personal Information section data elements of delivery register are completely recorded** | | | | | | | | | | | | | | | | | **Recorded** | | | | **Readable** | | | | |
|  |  |  |  |  |  |  |  |  |  |  |  |  |  |  |  |  |  |  |  |  |  | Yes**√)** | | | No **(√)** | Yes**√)** | | | No **(√)** | |
|  |  |  |  |  | a | | | Sequential serial number in registration book | | | | | | | | | | | | | |  | | |  |  | | |  | |
|  |  |  |  |  | b | | | Medical Record Number (MRN) | | | | | | | | | | | | | |  | | |  |  | | |  | |
|  |  |  |  |  | c | | | Name of the mother | | | | | | | | | | | | | |  | | |  |  | | |  | |
|  |  |  |  |  | d | | | Age of the mother | | | | | | | | | | | | | |  | | |  |  | | |  | |
|  |  |  |  |  | e | | | Kebele (Address) the mother | | | | | | | | | | | | | |  | | |  |  | | |  | |
| 102 | | | | | **Labor and maternal outcome section data elements of delivery register is completely recorded** | | | | | | | | | | | | | | | | | | | | | | | | | |
|  |  |  |  |  | a | | | Delivery Date | | | | | | | | | | | | | |  | | |  |  | | |  | |
|  |  |  |  |  | b | | | Delivery time | | | | | | | | | | | | | |  | | |  |  | | |  | |
|  |  |  |  |  | c | | | Mode of delivery: | | | | | | | | | | | | | |  | | |  |  | | |  | |
| 103 | | | | |  | | | Maternal status | | | | | | | | | | | | | |  | | |  |  | | |  | |
| 105 | | | | | New-born birth outcome section | | | | | | | | | | | | | | | | |  | | |  |  | | |  | |
|  |  |  |  |  | a | | | **Outcome**: | | | | | | | | | | | | | |  | | |  |  | | |  | |
|  |  |  |  |  | b | | | Apgar score | | | | | | | | | | | | | |  | | |  |  | | |  | |
|  |  |  |  |  | c | | | Sex of newborn | | | | | | | | | | | | | |  | | |  |  | | |  | |
|  |  |  |  |  | d | | | Weight in gram | | | | | | | | | | | | | |  | | |  |  | | |  | |
|  |  |  |  |  | e | | | TTC eye ointment | | | | | | | | | | | | | |  | | |  |  | | |  | |
|  |  |  |  |  | f | | | BCG Given | | | | | | | | | | | | | |  | | |  |  | | |  | |
|  |  |  |  |  | g | | | OPV0 Given | | | | | | | | | | | | | |  | | |  |  | | |  | |
| 106 | | | | | **Maternal HIV care and follow-up** | | | | | | | | | | | | | | | | | | | | | | | | | |
|  |  |  |  |  | a | | | Known HIV Positive | | | | | | | | | | | | | |  | | |  |  | | |  | |
|  |  |  |  |  | b | | | HIV test accepted (New or retest) | | | | | | | | | | | | | |  | | |  |  | | |  | |
|  |  |  |  |  | c | | | HIV test result | | | | | | | | | | | | | |  | | |  |  | | |  | |
|  |  |  |  |  | d | | | Counseled on breast feeding option | | | | | | | | | | | | | |  | | |  |  | | |  | |
| 107 | | | | | **Partner HIV Testing** | | | | | | | | | | | | | | | | | | | | | | | | | |
|  |  |  |  |  | a | | | Partner HIV test accepted | | | | | | | | | | | | | |  | | |  |  | | |  | |
|  |  |  |  |  | b | | | Partner HIV test result | | | | | | | | | | | | | |  | | |  |  | | |  | |
| 108 | | | | | | | | Name and signature | | | | | | | | | | | | | |  | | |  |  | | |  | |
| **Section 2: Completeness of data elements on IIF** | | | | | | | | | | | | | | | | | | | | | |  | | |  |  | | |  | |
| 201 | | |  | | | | **Front section of IIF** | | | | | | | | | **Recorded** | | | | | | | | | **Readable** | | | | |  |
|  |  |  |  |  |  |  |  |  |  |  |  |  |  |  |  | Yes**√)** | | | | No **(√)** | | | | | Yes**√)** | | No **(√)** | | |  |
|  |  |  | a | | | | Name of facility | | | | | | | | |  | | | |  | | | | |  | |  | | |  |
|  |  |  | b | | | | MRN of mother | | | | | | | | |  | | | |  | | | | |  | |  | | |  |
|  |  |  | c | | | | Full name of the mother | | | | | | | | |  | | | |  | | | | |  | |  | | |  |
|  |  |  | d | | | | Date of registration | | | | | | | | |  | | | |  | | | | |  | |  | | |  |
|  |  |  | e | | | | Sex | | | | | | | | |  | | | |  | | | | |  | |  | | |  |
|  |  |  | f | | | | Date of birth of mother/ or Age of mother | | | | | | | | |  | | | |  | | | | |  | |  | | |  |
|  |  |  | g | | | | Address of mother (If at least kebele is recorded) | | | | | | | | |  | | | |  | | | | |  | |  | | |  |
| 202 | | | **Inside section of IIF** | | | | | | | | | | | | | | | | | | | | | | | | | | | |
|  |  |  | a | | | | The current delivery date and time of the mother recorded | | | | | | | | |  | | | |  | | | | |  | |  | | |  |
|  |  |  | b | | | | Service: if the department providing service | | | | | | | | |  | | | |  | | | | |  | |  | | |  |
|  |  |  | c | | | | Serial number in service registration book | | | | | | | | |  | | | |  | | | | |  | |  | | |  |
| **Section 3: Completeness of data elements on integrated card** | | | | | | | | | | | | | | | | | | | | | | | | | | | | | |  |
| 301 | | | | Woman’s integrated card found filed within Woman’s IIF | | | | | | | | | | | | | | | 1.Yes 2. No | | | | | | | | | **If No skip to section 4** | | |
|  | | | | **Back ground information sections /Intrapartum Care and Follow up data elements** | | | | | | | | | | | | | | | | | | | | | | | |  | | |
|  | |  | | **Data Elements** | | | | | | | | **Recorded** | | | | | | | **Readable** | | | | | | | | |  | | |
|  |  |  |  |  |  |  |  |  |  |  |  | **Yes√)** | | | **No (√)** | | | | **Yes√)** | | | | | | **No (√)** | | |  | | |
|  |  | a | | Name of the mother | | | | | | | |  | | |  | | | |  | | | | | |  | | |  | | |
|  |  | b | | Gravida | | | | | | | |  | | |  | | | |  | | | | | |  | | |  | | |
|  |  | c | | Para | | | | | | | |  | | |  | | | |  | | | | | |  | | |  | | |
|  |  | d | | MRN | | | | | | | |  | | |  | | | |  | | | | | |  | | |  | | |
|  |  | e | | Date of Admission | | | | | | | |  | | |  | | | |  | | | | | |  | | |  | | |
|  |  | f | | Time of admission | | | | | | | |  | | |  | | | |  | | | | | |  | | |  | | |
|  |  | g | | Ruptured Membranes/ Hours | | | | | | | |  | | |  | | | |  | | | | | |  | | |  | | |
|  | | | | **Delivery Summary section of integrated card** | | | | | | | | | | | | | | | | | | | | | | | |  | | |
|  |  |  |  |  | | | | | | | **Recorded** | | | | | | | | | | **Readable** | | | | | | |  | | |
|  |  |  |  |  |  |  |  |  |  |  | **Yes√)** | | | | **No (√)** | | | | | | **Yes√)** | | | | **No (√)** | | |  | | |
|  | a | | | date of delivery | | | | | | |  | | | |  | | | | | |  | | | |  | | |  | | |
|  | b | | | time of delivery | | | | | | |  | | | |  | | | | | |  | | | |  | | |  | | |
|  | c | | | mode of delivery: | | | | | | |  | | | |  | | | | | |  | | | |  | | |  | | |
|  | d | | | placenta: | | | | | | |  | | | |  | | | | | |  | | | |  | | |  | | |
|  | e | | | laceration: | | | | | | |  | | | |  | | | | | |  | | | |  | | |  | | |
|  | f | | | newborn sex | | | | | | |  | | | |  | | | | | |  | | | |  | | |  | | |
|  | g | | | new born outcome | | | | | | |  | | | |  | | | | | |  | | | |  | | |  | | |
|  | h | | | apgar score | | | | | | |  | | | |  | | | | | |  | | | |  | | |  | | |
|  | i | | | birth weight | | | | | | |  | | | |  | | | | | |  | | | |  | | |  | | |
|  | j | | | length | | | | | | |  | | | |  | | | | | |  | | | |  | | |  | | |
|  | k | | | single/multiple | | | | | | |  | | | |  | | | | | |  | | | |  | | |  | | |
|  | l | | | term/preterm | | | | | | |  | | | |  | | | | | |  | | | |  | | |  | | |
|  | m | | | hiv testing offered | | | | | | |  | | | |  | | | | | |  | | | |  | | |  | | |
|  | n | | | hiv testing accepted | | | | | | |  | | | |  | | | | | |  | | | |  | | |  | | |
|  | o | | | hiv test result recorded | | | | | | |  | | | |  | | | | | |  | | | |  | | |  | | |
|  | p | | | feeding option recorded | | | | | | |  | | | |  | | | | | |  | | | |  | | |  | | |
|  | q | | | name and signature | | | | | | |  | | | |  | | | | | |  | | | |  | | |  | | |
|  |  |  |  |  |  |  |  |  |  |  |  |  |  |  |  |  |  |  |  |  |  |  |  |  |  |  |  |  |  |  |
|  | | | **Section 4: Consistency of data elements among delivery register, IIF, and Integrated card** | | | | | | | | | | | | | | | | | | | | | | | | | | | |
|  | | | **Data elements** | | | | | | **Consistently Recorded** | | | | | | | | | | | | | | | | | | | | | |
|  |  |  |  |  |  |  |  |  | IIF | | | | | | | | Integrated card | | | | | | | | | | | | | |
|  |  |  |  |  |  |  |  |  | **Yes√)** | | | | **No (√)** | | | | **Yes√)** | | | | | | **No (√)** | | | | | | | |
| 401 | | | a | | | Serial number | | | |  | | | |  | | | |  | | | | | |  | | | | | | |
|  |  |  | b | | | MRN | | | |  | | | |  | | | |  | | | | | |  | | | | | | |
|  |  |  | c | | | Full name of the mother | | | |  | | | |  | | | |  | | | | | |  | | | | | | |
|  |  |  | f | | | Delivery Date | | | |  | | | |  | | | |  | | | | | |  | | | | | | |
|  |  |  | g | | | Delivery time | | | |  | | | |  | | | |  | | | | | |  | | | | | | |
|  |  |  | h | | | Mode of delivery | | | |  | | | |  | | | |  | | | | | |  | | | | | | |
|  |  |  | i | | | Apgar score | | | |  | | | |  | | | |  | | | | | |  | | | | | | |
|  |  |  | j | | | Sex of newborn | | | |  | | | |  | | | |  | | | | | |  | | | | | | |
|  |  |  | k | | | Newborn outcome | | | |  | | | |  | | | |  | | | | | |  | | | | | | |
|  |  |  | l | | | Weight in gram | | | |  | | | |  | | | |  | | | | | |  | | | | | | |
|  |  |  | m | | | HIV test accepted (Yes or No) | | | |  | | | |  | | | |  | | | | | |  | | | | | | |
|  |  |  | n | | | HIV test result ((Yes or No)) | | | |  | | | |  | | | |  | | | | | |  | | | | | | |
|  |  |  | o | | | Name and signature | | | |  | | | |  | | | |  | | | | | |  | | | | | | |
